# Supplementary material for: Unplanned hospital visits after ambulatory surgical care
Source: PLoS One. 2021 Jul 20;16(7):e0254039. doi: 10.1371/journal.pone.0254039 (PMC8291649; doi:10.1371/journal.pone.0254039)
Supplement: S1 Appendix — ICD-9-CM, International Classification of Diseases, Ninth Revision, Clinical Modifications. (DOCX) [file pone.0254039.s001.docx]

**Appendix I. ICD-9-CM coding appendix**

| *Term* | *ICD-9-CM codes* |
| --- | --- |
| Maintenance radiation or chemotherapy | V58.0, V58.1, V58.11, V58.12, V66.1, V66.2, V67.1, V67.2 |
| Rehabilitation services | V52.0, V52.1, V52.4, V52.8, V52.9, V53.8, V57.0, V57.1, V57.2, V57.21, V57.22, V57.3, VS5.4, V57.81, V57.89, V57.9, V58.82 |
| Cancer | 140.x-239.x |
| Normal obstetrical delivery | 650, 651.00, 651.01, 651.10, 651.11, 651.20, 651.21, 651.70, 651.71, 651.73, 651.80, 651.81, 651.90, 651.91  V22.0, V22.1, V22.2, V24.1, V24.2, V27.0, V27.1, V27.2, V27.3, V27.4, V27.5, V27.6, V27.7, V27.9, V72.4, V72.42, V91.11, V91.12, V91.19, V91.20, V91.21, V91.22, V91.29, V91.90, V91.91, V91.92, V91.99 |

*ICD-9-CM*, International Classification of Diseases, Ninth Revision, Clinical Modifications.
